# Functional interplay between the RK motif and linker segment dictates Oct4–DNA recognition

**DOI:** 10.1093/nar/gkv323

**Published:** 2015-04-13

**Authors:** Xiangqian Kong, Jian Liu, Lianchun Li, Liyan Yue, Lihong Zhang, Hualiang Jiang, Xin Xie, Cheng Luo

**Affiliations:** 1 Drug Discovery and Design Center, State Key Laboratory of Drug Research, Shanghai Institute of Materia Medica, Chinese Academy of Sciences, Shanghai 201203, China; 2 Chinese Academy of Sciences Key Laboratory of Receptor Research, National Center for Drug Screening, Shanghai Institute of Materia Medica, Chinese Academy of Sciences, Shanghai 201203, China

## Abstract

The POU family transcription factor Oct4 plays pivotal roles in regulating pluripotency and somatic cell reprogramming. Previous studies have indicated an important role for major groove contacts in Oct4–DNA recognition; however, the contributions of the RK motif in the POUh domain and the linker segment joining the two DNA-binding domains remain poorly understood. Here, by combining molecular modelling and functional assays, we find that the RK motif is essential for Oct4–DNA association by recognizing the narrowed DNA minor groove. Intriguingly, computational simulations reveal that the function of the RK motif may be finely tuned by H-bond interactions with the partially disordered linker segment and that breaking these interactions significantly enhances the DNA binding and reprogramming activities of Oct4. These findings uncover a self-regulatory mechanism for specific Oct4–DNA recognition and provide insights into the functional crosstalk at the molecular level that may illuminate mechanistic studies of the Oct protein family and possibly transcription factors in the POU family. Our gain-of-function Oct4 mutants might also be useful tools for use in reprogramming and regenerative medicine.

## INTRODUCTION

Oct4 lies at the centre of the core regulatory network governing pluripotency and somatic cell reprogramming (1–3). Both the knockdown and overexpression of Oct4 in embryonic stem cells (ESCs) result in cellular differentiation, suggesting that tight regulation of Oct4 expression level is essential for maintaining pluripotency (4–6). Oct4 belongs to the POU (Pit-Oct-Unc) transcription factor family. Members of this family bind to the consensus octamer motif AGTCAAAT through bipartite DNA-binding domains (POU domain) and modulate target gene expression by both N- and C-terminal transactivation domains (7–9).

The POU domain consists of two structurally independent subdomains—the POU-specific domain (POUs) and the POU homeodomain (POUh)—connected by a linker of variable length and composition (7–8,10). Previous studies have emphasized the importance of the helix-turn-helix (HTH) elements of POUs and POUh subdomains in the recognition of specific DNA sites by Oct4 and other POU family members (11–13). The HTH motif employs a base readout mechanism that involves insertion into the major groove of DNA and forming extensive hydrogen bond (H-bond) or hydrophobic contacts with bases. Disruption of these conserved interactions greatly reduces the DNA binding affinity of POU family members (14,15).

In addition to making contact with the major groove, the N-terminal arm of the POUh subdomain—a cluster of positively charged residues designated as RK motif—has been suggested to mediate minor groove interactions by tracing along the minor groove at the octamer site (11–12,15). In most POU family proteins, the majority of RK-motif residues mediate H-bond and electrostatic interactions with bases and the DNA backbone. However, the sidechains of the Oct4 RK motif are largely disordered (with the exception of R227), and only two H-bond contacts are formed between the RK motif and DNA sequences in the Oct4–DNA complex (12). These observations suggest that the RK motif is dispensable for Oct4–DNA recognition. However, multiple sequence alignment of POU family members and Oct4 orthologues reveals that the RK motif is highly conserved, including the residues with disordered sidechains in Oct4 (Supplementary Figure S1A and B), suggesting an important function of this motif. Moreover, the *Drosophila* POU protein I-POU, which lacks two basic residues in the RK motif, is incapable of binding to any DNA sequence. The introduction of two basic amino acids into the RK motif restored its DNA binding properties, implicating the non-trivial role of the RK motif in the DNA binding (16,17). These controversial lines of evidence call into question the importance of the RK motif for specific DNA recognition.

In contrast to the conserved POUs and POUh subdomains, the linker segment connecting these subdomains exhibits very low sequence conservation (Supplementary Figure S1A). In most POU protein–DNA complex structures, unstructured or partially visible linker segments do not come into direct contact with the target DNA (18). Studies of Oct1–DNA binding suggest that the linker may contribute favourably to the energetics of DNA binding through chelation, thereby maintaining a high local concentration of subdomains around the DNA site and increasing their binding to DNA. The length of the linker may also regulate the configurational freedom of the two structured subdomains relative to the DNA sites, thereby influencing DNA site selection via a tethering mechanism (19). In Oct4–DNA complex structure, a short α-helix is observed in the partially disordered linker segment (residues from N206 to A222), in which six residues have a disordered sidechain and four residues are completely disordered. The functional study showed that the short α-helix in the linker segment of Oct4 could modulate its reprogramming potential by serving as a protein–protein interaction (PPI) site. Mutations at the unique interface completely abolish the reprogramming potential but have minimal effects on DNA binding (12). Despite our increased understanding of the role played by linker segments in regulating Oct4–DNA association and transactivation, little attention has been paid to the impact of the partially disordered linker segment's sequence composition on DNA binding properties and the underlying mechanisms.

Here, by employing molecular dynamic (MD) simulations and functional assays with various Oct4 mutants, we show that the RK motif and its crosstalk with the linker segment are of great importance for Oct4–DNA binding. The clustered positive charges and unique structural features observed in MD simulations may enable the RK motif to use a shape-based readout mechanism to detect the enhanced negative electrostatic potential in the minor grove of Oct4 recognition sites. Our mutagenesis data demonstrated that the DNA binding capability of Oct4 decreased progressively with the stepwise removal of positive charge from the RK motif. Furthermore, the MD simulations suggested that H-bond interactions between the disordered region in the linker segment and the RK motif may finely tune the DNA association of Oct4 and that the disruption of these interactions enhanced the DNA binding affinity, transactivation potential and somatic cell reprogramming efficiency of OCT4. Our results revealed a novel regulatory mechanism underlying Oct4–DNA recognition and underlined the relevance of the interplay between the RK motif and linker segment in somatic cell reprogramming.

## MATERIALS AND METHODS

### REMD and MD simulations

Sixty-four replicas spanning a temperature range of 300–390 K were used in the replica exchange molecular dynamics (REMD) simulations involving apo-Oct4 and each replica was simulated for 400 ns. Three independent 200-ns MD simulations were performed for four systems with the DNA conformation from the Oct4–H2B complex, Oct4–PORE complex and 3DNA-generated models for both H2B and PORE. All of the MD and REMD simulations were carried out using the GROMACS 4.5.3 package (20,21). The Curves+ (22,23) and Delphi programs (24–26) were used to calculate the minor groove width and electrostatic potential as previously described (27,28). The details of the computational simulations are provided in the Supporting Information (SI).

### Electrophoretic mobility shift assay

The probe-protein mixtures were electrophoresed on a native polyacrylamide gel (5%). The intensity of the DNA bands was then visualized and quantified after staining.

### Luciferase reporter assay

Constructs encoding HA-Oct4 (WT or mutant), firefly luciferase reporter under control of the TK promoter and 6W enhancer and the control Renilla luciferase reporter (pRL-TK) were transfected into HEK293T cells using Fugene transfection reagent (Life Technologies). Firefly and Renilla luciferase activities were measured after 24 h with the Dual-Glo Luciferase Assay System (Promega) on an EnVision multilabel plate reader (Perkin Elmer). Firefly luciferase activity was normalized to Renilla luciferase activity for each sample.

### Mouse iPSC generation

Mouse iPSCs were generated using pMXs retroviral vectors encoding mouse Oct4 (WT or mutant), Sox2 and Klf4. Briefly, MEFs carrying an Oct4–GFP reporter were infected with viruses containing 3F and re-seeded at a density of 10 000 cells per well onto 24-well plates pre-seeded with feeders (SI Materials and Methods). GFP^+^ colonies were photographed and counted at day 16 with an Olympus microscope using Image Pro Plus software.

### Statistical analysis

Values are reported as means ± SEM and were analysed using the two-tailed Student's *t*-test. P < 0.05 was considered statistically significant.

### Additional details

The remaining experimental details are given in SI Materials and Methods.

## RESULTS

### The RK-motif is flexible and solvent-accessible in apo-state Oct4

**Figure 1.**
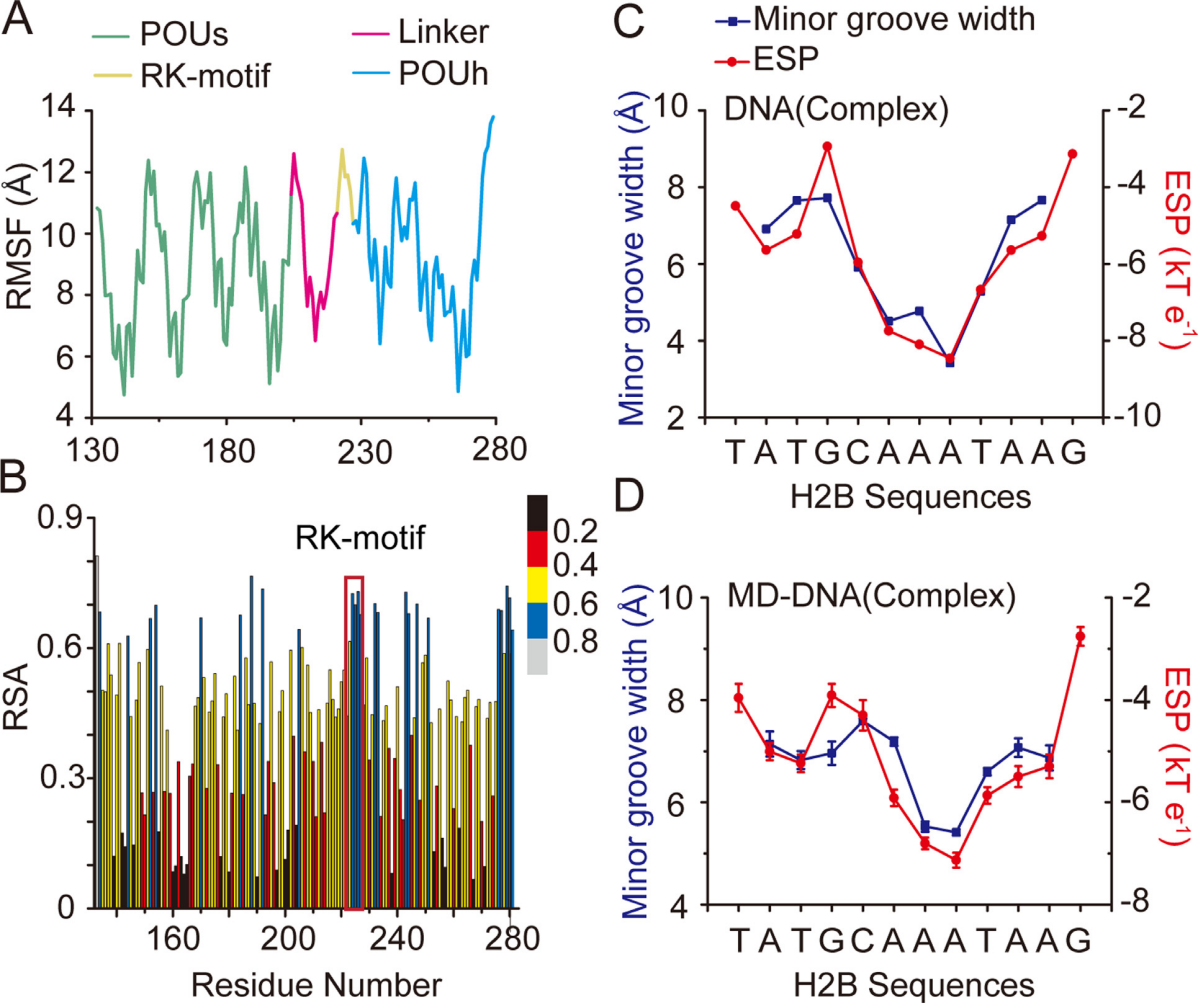
The structural properties of RK-motif and Oct4 recognition sites. (**A**) Residue fluctuations obtained by averaging residual fluctuations over the REMD simulations of apo-state Oct4. (**B**) The relative solvent accessibility of each residue in REMD simulations of apo-state Oct4 and the RK-motif is highlighted. (**C**) The minor groove width and electrostatic potential (ESP) of the H2B site in the modelled Oct4–DNA complex. (**D**) The minor groove width and ESP of the H2B site in an MD simulation with the initial DNA conformation from the modelled Oct4–DNA complex. The data (means ± SD) were obtained from three independent MD simulations.

Comparisons between the structures of DNA-binding proteins in the presence and absence of DNA may help to identify the key elements and mechanisms underlying dynamic protein–DNA recognition (29,30). However, structural data are currently unavailable for apo-state Oct4 and other POU family members. To gain insight into the conformational space and structural features of Oct4 in the absence of DNA, the REMD method is employed in our study, with the initial conformation from the Oct4–DNA complex (PDB entry: 3L1P) (12). As an enhanced sampling approach compared to traditional MD simulations, REMD allows systems to escape from local energy traps by exchanging replicas concurrently simulated at different temperatures based on the Metropolis criterion (31,32). The large and uniform acceptance ratios (0.25 ± 0.01; Supplementary Table S1), as well as a substantial overlap of canonical probability distributions between all neighbouring pairs of temperatures (Supplementary Figure S1C), indicated that a sufficient number of replica exchanges between pairs of replicas could be achieved (31). These data are consistent with the observed random walk of the replica within the simulated temperature space (Supplementary Figure S1D). Consequently, all evidence suggests that a sufficient number of replicas were properly distributed in the temperature space and that the REMD simulations were performed properly and effectively. To understand the structural characteristics of apo-Oct4 in solution, we categorized the conformations sampled at a physiologically relevant temperature (300 K) based on structural similarity. The relative arrangement of the two subdomains (Supplementary Figure S2A), rather than the deformation of subdomain architecture, accounted for most of the significant conformational differences between apo-Oct4 and Oct4 present in an Oct4–DNA complex (Supplementary Figure S2B). These results are in agreement with experimental observations, which suggest that a remarkable conformational change is required for Oct1–DNA association even though its two subdomains maintain rigid folding in solution (19,33). Nevertheless, the highly conserved RK-motif in the POUh subdomain exhibits recurring structural features in REMD simulation. The root-mean-square ﬂuctuation (RMSF), which represents the average displacement of residues throughout the simulation process, reflects the fact that the RK-motif has a high flexibility similar to the inter-helical loops in each of the subdomains (Figure 1A and Supplementary Figure S2A). Moreover, solvent accessibility analysis demonstrates that the positively charged RK-motif is highly solvent-accessible in solution, a prerequisite for bio-molecular interactions (34) Figure 1B). Collectively, the REMD simulations reveal that the RK-motif, which consists solely of positively charged residues that are highly conserved across both paralogues (Supplementary Figure S1A) and orthologues (Supplementary Figure S1B) of Oct4, has a remarkable flexibility and solvent accessibility in apo-state Oct4.

### Minor groove narrowing and enhanced negative electrostatic potential are intrinsic properties of Oct4 recognition sites

Recent studies emphasize the importance of the unique three-dimensional structure of DNA and local variations in its topography for protein–DNA recognition (27–28,35–37). An AT-rich motif is observed within the binding sites of most Oct4-regulated genes (38). Multiple lines of evidence have revealed that the narrow minor grooves are often associated with the presence of AT-rich motifs termed ‘A-tracts’, which consist of runs of at least three consecutive ApA, TpT or ApT base pair steps (39,40). Rohs *et al*. further showed that the narrow width of the minor groove substantially enhances the negative electrostatic potential around the DNA motif, which plays essential roles in the specific recognition of DNA-binding proteins (27,35–36). Hence, to explore this unique structural characteristic within the context of Oct4 recognition sites, we calculated both the minor groove widths and electrostatic potentials of two A-tract-containing motifs (H2B and PORE) occupied by Oct4 both *in vitro* and *in vivo*. (41) As illustrated in Figure 1C and Supplementary Figure S3A, minor grooves as well as enhanced negative electrostatic potentials were clearly observed around the A-tracts in both the H2B and pseudo-palindromic PORE motifs in comparison with those of the ideal B-form double-helix models generated by the 3DNA program (Supplementary Figure S3B and C) (42).

To understand the dynamic behaviour of the Oct4 recognition sites, we performed MD simulations for the H2B and PORE sequence motifs with two different initial conformations for each. One conformation was extracted from the Oct4–DNA complex, whereas the other was the corresponding 3DNA-generated model for the ideal B-form double helix. Monitoring the minor groove width and electrostatic potential along each trajectory reveals that the trends of minor groove narrowing and enhanced electrostatic potential are maintained at H2B and PORE sites, as reflected by the global minimums of these properties at the A-tracts (Figure 1D and Supplementary Figure S3D). Intriguingly, when the ideal B-form models were taken as initial conformations, we observed highly similar profiles (Supplementary Figure S3E and F) to those employing the initial configurations from the protein–DNA complex (Figure 1D and Supplementary Figure S3D). Thus, these data collectively implicate minor groove narrowing and enhanced negative electrostatic potential as intrinsic properties of Oct4 recognition sites containing A-tracts.

### Mutations in the RK motif impair Oct4 DNA binding affinity, transactivation potential and reprogramming efficiency

**Figure 2.**
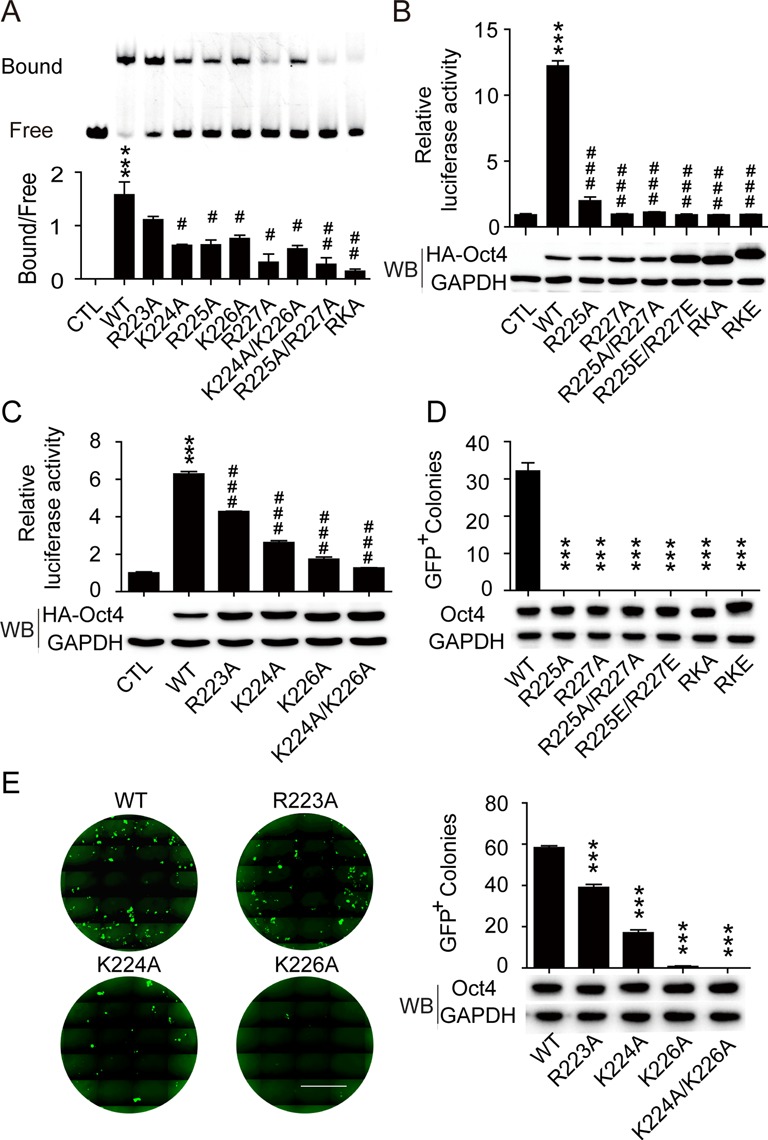
Mutation of the RKRKR motif disrupts the Oct4–DNA interaction. (**A**) WT and RKRKR motif mutant Oct4 were assessed for their ability to bind to DNA sequences containing a single octamer motif with an electrophoretic mobility shift assay. A representative gel is presented at top and a statistical analysis of the bound and free forms of DNA is presented at bottom. Data are presented as means ± SEM (*n* = 3). ***P < 0.001 versus control; ^#^*P* < 0.05, ^##^*P* < 0.01 versus WT Oct4. (**B**) and (**C**) (Top) The transcriptional activities of WT and mutant Oct4 were measured using a luciferase reporter containing an Oct4-driven enhancer. Luciferase activity was measured 24 h after transfection and the control group was transfected with the reporter only. (Bottom) Corresponding exogenous Oct4 protein levels were measured by western blot. Data are means ± SEM (*n* = 3). ***P < 0.001 versus control; ^###^*P* < 0.001 versus WT Oct4. (**D**) (Top) The number of GFP-positive iPSC colonies generated with three factors (WT or mutant Oct4, Sox2 and Klf4) at day 16 following infection. (Bottom) Corresponding exogenous Oct4 protein levels were measured on day 3 following infection by western blot. (**E**) (Left) Representative images of iPSC colonies; scale bar: 5 mm. (Right) The numbers of GFP-positive iPSC colonies generated with three factors (WT or mutant Oct4, Sox2 and Klf4) at day 16 following infection. The corresponding exogenous Oct4 protein levels were measured on day 3 by western blot. Data are presented as means ± SEM (n = 3). ***P < 0.001 versus WT Oct4.

The molecular modelling data suggested that the flexible and solvent-exposed nature of the RK-motif might facilitate interactions between its positively charged residues and the minor groove at Oct4 recognition sites that are characterized by enhanced negative electrostatic potentials, which may thus be important in Oct4–DNA association. To test this hypothesis, we compared the DNA binding ability of Oct4 variants with mutations in the RK motif with an electrophoretic mobility shift assay (43). As shown in Figure 2A, replacing any of the lysine or arginine residues (except for R223) in the RK motif with alanine significantly reduced Oct4–DNA binding. The further decreased positive charges in K224A/K226A and R225A/R227A double mutants may account for the greater reduction observed in their DNA binding capabilities. The substitution of all residues in the RK-motif with alanine (RKA) almost completely abolished Oct4–DNA binding (Figure 2A). These data highlight the importance of each residue in the RK-motif for Oct4–DNA binding, which may form contacts via the enhanced electrostatic potential of the minor groove.

The transcriptional function of the Oct4 mutants was tested with a luciferase reporter assay using six copies of octamer-containing oligonucleotide (6W) as an enhancer (44). All point mutations in the RK-motif displayed a detrimental impact on the transactivation activity of Oct4, with R223A being the least affected and R227A the most affected (Figure 2B and C). Only basal levels of activity were observed in Oct4s carrying two or more mutations (K224A/K226A, R225A/R227A, RKA and RKE), corresponding with their reduced DNA binding capabilities. Next, we investigated these RK-motif mutants in 3F (Oct4, Sox2 and Klf4)-mediated somatic cell reprogramming. The R223A and K224A mutants were able to generate iPSCs with reduced efficiency, but the R225A, K226A and R227A single site mutants and the double or multiple site mutants (K224A/K226A, R225A/R227A, RKA and RKE) lost the ability to generate iPSCs almost completely (Figure 2D and E). The greatly diminished reprogramming efficiency of these RK-motif mutants may result from the attenuated occupancy of Oct4 at pluripotency-related target genes (Oct4, SOX2 and JARID2), as demonstrated in a chromatin immunoprecipitation (ChIP) assay (Figure 4E), consistent with their reduced DNA binding capabilities. Because comparable levels of the mutant proteins were detected in these assays, the loss-of-function phenotype should not be attributed to differential expression between the mutants. Taken together, our data indicate that all of the residues, including those invisible in the Oct4–DNA complex structure, play indispensable roles in the DNA binding, transactivation and reprogramming functions of Oct4.

**Figure 3.**
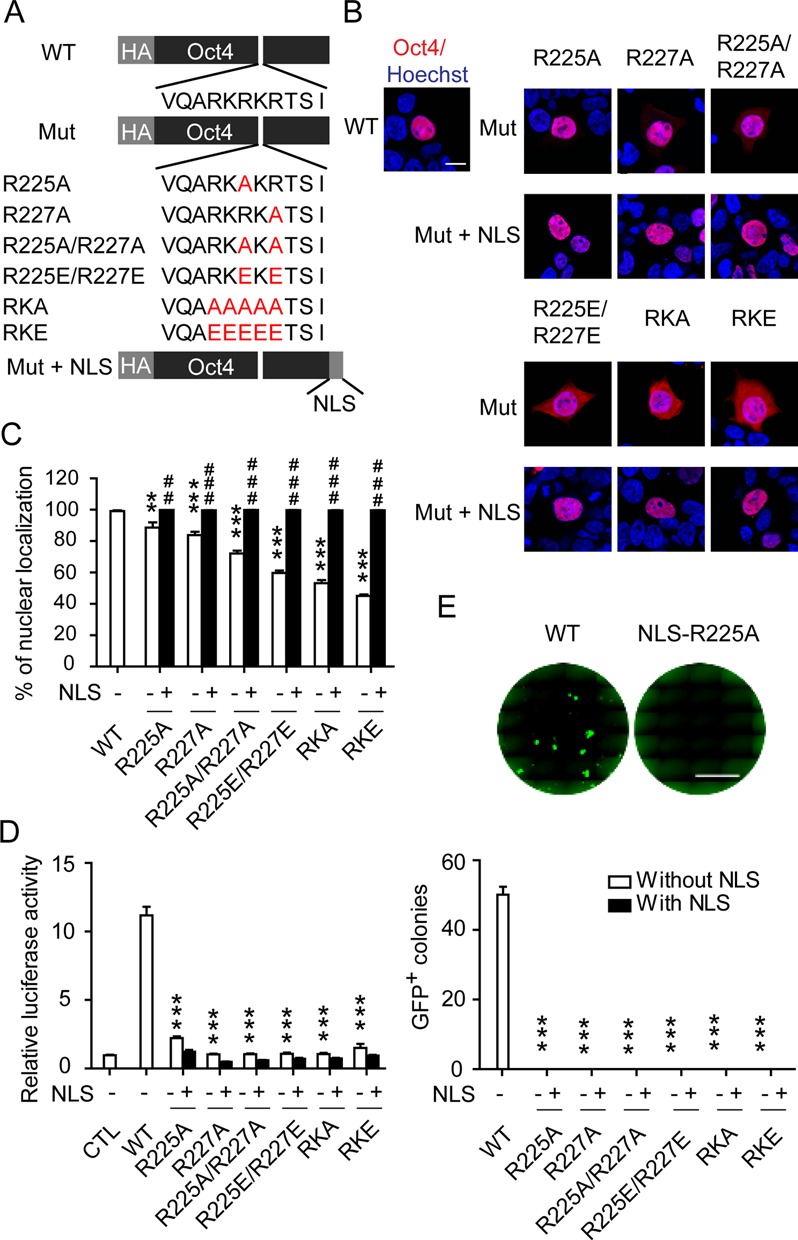
The RKRKR motif mutants of Oct4 lose transcriptional activity independent of localization. (**A**) Schematic diagram of the Oct4 mutants at the RKRKR motif with or without an additional nuclear localization sequence (NLS). (**B**) Cellular localization of Oct4 mutants with or without an additional NLS. Immunofluorescence staining with an anti-HA antibody was carried out 48 h after transfection in HEK293 cells. Hoechst stain was used to mark the nuclei. Scale bar: 10 μM. (**C**) Statistical analysis of the nuclear localization of various Oct4 proteins presented in (B). Data are presented as means ± SEM (*n* = 10). ***P < 0.001 versus WT Oct4; ^###^*P* < 0.001 versus corresponding mutants without an additional NLS at the C terminal. (**D**) The transcriptional activities of WT and mutant Oct4 with or without an additional NLS were measured by luciferase reporter assay. Luciferase activities were measured 24 h after transfection and the control group was transfected with the reporter only. Data are presented as means ± SEM (*n* = 3). ***P < 0.001 versus WT Oct4. (**E**) iPSCs were induced with three factors (various Oct4 mutant, Sox2 and Klf4) and the number of GFP-positive colonies was counted at day 16 following infection. Representative images of iPSC colonies are presented; scale bar: 5 mm. Data are presented as means ± SEM (*n* = 3). ***P < 0.001 versus WT Oct4.

**Figure 4.**
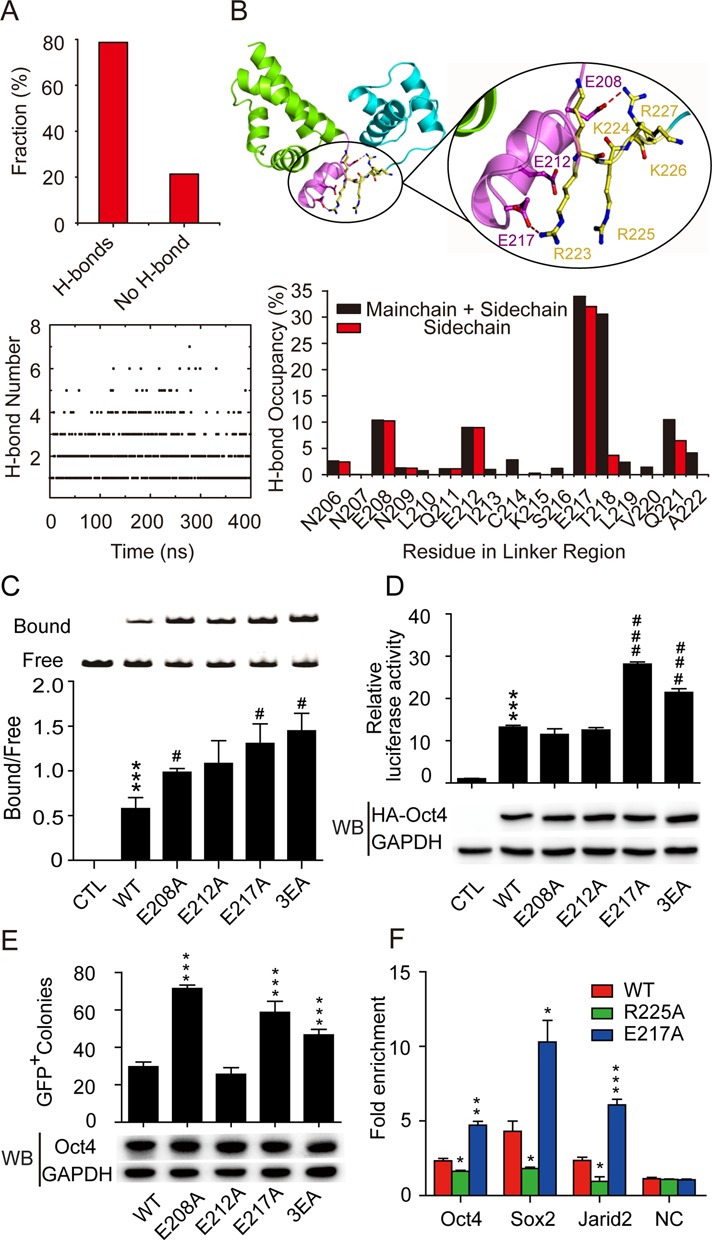
Interaction of the RKRKR motif and the linker determines Oct4–DNA recognition and binding. (**A**) The fraction of the H-bond interactions (Top) and the time series of H-bond number (Bottom) between the RK-motif and Linker segment during the REMD simulations. (**B**) A representative conformation showing the hydrogen-bond interactions between the RK motif and the linker region (top) and the H-bond occupancy statistics for each residue in the linker region (bottom) during REMD simulations. (**C**) (Top) A representative gel from the electrophoretic mobility shift assay to assess the binding of mutant Oct4 at the linker region with DNA. (Bottom) Statistical analysis of bound and free forms of DNA. Data are means ± SEM (*n* = 3). ***P < 0.001 versus control; ^#^*P* < 0.05. (**D**) (Top) The transcriptional activities of Oct4 mutants at the linker region were measured using a luciferase reporter assay. Luciferase activities were measured 24 h after transfection and the control group was transfected with the reporter only. (Bottom) Corresponding exogenous Oct4 protein levels were measured using Western blot. Data are means ± SEM (*n* = 3). ***P < 0.001 versus control; ^###^*P* < 0.001 versus WT Oct4. (**E**) (Top) iPSCs were induced with three factors (various Oct4 linker mutants, Sox2 and Klf4) and the numbers of GFP-positive colonies were counted at day 16 following infection. (Bottom) Corresponding exogenous Oct4 protein levels were measured on day 3 after infection using western blot. Data are means ± SEM (*n* = 3). ***P < 0.001 versus WT Oct4. (**F**) E14 mESCs were transfected with WT, R225A or E217A mutant Oct4, and their binding at the Oct4, Sox2 and Jarid2 loci was detected by ChIP analysis with anti-Oct4 antibody. Data are shown as fold enrichment relative to the IgG ChIP results. Data are presented as means ± SEM (*n* = 3). *P < 0.05, **P < 0.01, ***P < 0.001 versus WT Oct4. NC, negative control (a random DNA sequence).

### The impaired transcriptional activities of Oct4 RK-motif mutants are independent of nuclear localization

Previous studies have identified the RK-motif as a nuclear localization signal (NLS) for Oct4 and mutation of the RK motif results in a random distribution of Oct4 throughout the cell (45). Immunofluorescence staining revealed that single point mutations (R225A, R227A) had very limited impact on the nuclear localization of Oct4 (Figure 3A–C). However, these mutants were almost completely defective in transactivating the 6W-luciferase reporter and in generating iPSCs (Figure 3D and E). Similarly, the 30–60% reduction in nuclear localization of Oct4s carrying double or multi-site mutations (R225A/R227A, R225E/R227E, RKA and RKE) could not account for the total ablation of its transactivation and reprogramming capabilities. This finding suggests that the RK motif not only functions as an NLS but also strongly regulates the transcriptional activity of Oct4 by mediating Oct4–DNA binding. Nevertheless, to unambiguously define the role of the RK motif in modulating the transcription activity of Oct4, a known NLS was fused to the C-terminus of each Oct4 mutant to restore their nuclear localization (Figure 3A). The exogenous NLS indeed re-directed the RK-motif mutant proteins exclusively to the nuclei (Figure 3A–C), as previously observed (45). However, the restoration of nuclear localization did not rescue the abolished transactivation and reprogramming functions, and virtually no improvement in transcriptional activity was obtained for the Oct4 mutants with an exogenous NLS (Figure 3D and E). Notably, similar expression levels of the mutant Oct4 proteins were observed in these assays (Supplementary Figure S4). Therefore, our data establish that the transcriptional regulatory function of the RK-motif is independent of its nuclear localization activity.

### Mutations in the linker segment increase DNA binding and other functions of Oct4

Recent studies have emphasized the importance of disordered regions within transcription factors in regulating DNA recognition through transient and dynamic contact with the DNA binding interface (29,46–48). Although the linker segment displays low homology among members of the POU family, it contains an unusual number of polar residues (Supplementary Figure S1A). More specifically, three negatively charged residues that are present in and conserved between orthologues of Oct4 (Supplementary Figure S1B) are partially (E208 and E212) or totally (E217) disordered within the Oct4–DNA complex (12). Because these negatively charged residues are in the vicinity of the positively charged RK-motif, we investigated their H-bond interactions in REMD simulations. At least one H-bond was identified between the linker segment and RK-motif in 80% of all sampled conformations according to H-bond occupancy analysis (Figure 4A). Most conformations were predicted to form 1, 2 or 3 H-bonds, which accounted for over 85% of snapshots with H-bonds formed (Figure 4A bottom panel). To further map the molecular determinants of H-bond contacts, we individually investigated interactions with the side chains of the RK motif for each residue in the linker segment. As shown in Figure 4B, the three negatively charged residues (E208, E212 and E217) are most frequently involved in contacts with the positively charged RK motif, mainly through inter-side chain H-bond interactions. Among them, E217, invisible in the Oct4–DNA complex structure but highly conserved within Oct4 orthologues (Supplementary Figure S1B), has the highest H-bond occupancy in the linker segment. In addition to these glutamic acid residues, T218 and Q221, which also have relatively high H-bond occupancies, participate in H-bond contacts largely via their main chains. These data suggest that H-bond interactions between linker and RK motif might shield the positive charges on the RK motif from recognizing the minor groove electrostatic potential of the Oct4 binding site. Disruption of these H-bonds may thus facilitate Oct4–DNA binding and transactivation.

To test this hypothesis, we performed DNA binding experiments with Oct4s carrying mutations in the linker segment (E208, E212 or E217). As expected, E208A and E217A enhanced protein–DNA binding, whereas E212A showed an affinity for the binding site comparable to that of WT Oct4 (Figure 4C). An additive effect was observed for E208A/E212A/E217A (3EA) triple-mutant Oct4, for which the DNA binding affinity was further increased. Correspondingly, the transactivation potential and reprogramming efficiency of Oct4 were significantly elevated in E217A and 3EA mutants (Figure 4D and E). Furthermore, the ChIP assay indicated the increased occupancy of E217A mutant Oct4 at the pluripotency genes, which may contribute to its augmented reprogramming efficiency (Figure 4F). The comparable DNA binding potencies may account for the indistinguishable transactivation and reprogramming efficiencies observed for the E212A mutant and WT Oct4. The E208A Oct4 mutant transactivated the luciferase reporter with a potency similar to that of WT protein, possibly due to its moderately increased DNA binding affinity (Figure 4C and D). Surprisingly, this mutant displayed a significantly enhanced reprogramming efficiency (Figure 4E). Although the exact mechanism of this phenomenon remains unknown, we speculate that E208 might regulate interactions with other transcription factors or transcription machinery, thereby affecting reprogramming efficiency (12). Sequencing of genomic DNA from the iPSC lines confirmed the integration of the Oct4 mutations (Supplementary Figure S5A). The ESC-like morphology and immunofluorescence staining patterns of typical pluripotency markers (Supplementary Figure S5B), as well as the formation of teratomas *in vivo* (Supplementary Figure S5C), validated the authenticity of the iPSCs generated via linker segment mutation of Oct4. Collectively, these mutagenesis results reveal that the linker segment may regulate Oct4–DNA recognition through functional interplay with the RK-motif and breaking the H-bond interactions between them leads to enhanced Oct4–DNA binding affinity, transactivation potential and reprogramming efficiency.

## DISCUSSION

Specific protein–DNA recognition is essential to many fundamental biological processes (49–51). In addition to the widely accepted base readout mechanism involving contacts between a protein and the unique chemical signatures of DNA bases in the major groove, the shape readout mechanism by which the protein recognizes sequence-dependent deformations of the DNA helix also plays an important role in regulating target sequence selectivity (27–28,52). Recent studies have discovered that the functional specificity of DNA binding proteins mediates the dynamic and transient contacts between disordered regions of proteins and their DNA binding interfaces (29,53).

As an essential regulator of stem cell pluripotency, the functions of the POU transcriptional factor Oct4 rely on the binding of the POU domain to a variety of regulatory DNA sites (2). In contrast to the well-characterized contacts with bases in the major groove, little attention has been paid to the role of minor groove recognition by the RK motif of Oct4 and other POU family members, presumably due to the limited interactions and smaller buried surface area when compared to the major groove. In particular, the partially disordered structural features of the side chains in the RK motif of the Oct4–DNA complex raise further questions regarding its importance for specific DNA recognition (12). Furthermore, in addition to being a covalent linkage that increases the local concentration of subdomains around DNA, the regulatory functions of the linker segment and its possible crosstalk with other structural elements in Oct4–DNA association are poorly understood.

Our MD simulations of Oct4 in the apo state and its A-tract-containing recognition sites suggested that Oct4 might utilize the positively charged lysine and arginine residues in the dynamic and solvent-accessible RK motif to detect the enhanced negative potential of the minor groove. Like other proteins, especially the homodomain containing transcriptional factors, these interactions may be critical for Oct4–DNA binding.(27–28,54) Our mutagenesis and *in vitro* binding experiments established that each of the residues in the RK-motif is amenable to DNA recognition by Oct4. Consistent with these findings, the decreased DNA binding affinity caused by RK-motif mutation leads to the attenuated occupancy at pluripotency gene loci (Figure 4E) as well as a marked loss of both transactivation and somatic cell reprogramming activity. It should be noted that, apart from the electronegative potential, other important factors may also associated with the narrowed minor groove and thus contribute to Oct4–DNA binding. Previous studies showed that a more ordered, multi-layered hydration network was observed in the narrowed DNA minor grooves. (55,56) A larger entropy gain from the release of the ordered water would thus be achieved upon protein binding.(57) The impaired DNA binding affinity after mutation of the positively charged residues inserted into the minor grooves, such as R227 in Oct4, may also be attributed to the decreased entropy gain, in addition to the reduced electrostatic attractions. Moreover, the narrowed minor groove may create a unique hydrophobic environment due to the propeller twisting, which results in the formation of inter base pair hydrogen bonds in the major groove.(58) Although the functional importance of these factors in Oct4–DNA binding is largely unknown, further investigation would provide us an in-depth understanding of the other mechanism in shape-based readout. Nevertheless, our results corroborate the crucial role of minor groove shape-based readout by the RK-motif in Oct4–DNA recognition.

The functional importance of the RK motif prompted us to explore the intramolecular contacts that might finely tune DNA recognition by Oct4. Our H-bond occupancy analysis revealed that negatively charged residues in the adjacent linker segment form H-bond interactions with the RK motif in most of the sampled conformations. Because in most cases three H-bonds or fewer are formed, it is likely that these interactions partially shield the positive charges of the RK motif and fine tune its ability to sense the unique minor groove electrostatic potential. Consistent with this hypothesis, disruption of these H-bonds by mutagenesis increased the DNA binding affinity of the E217A mutant and 3EA triple-mutant Oct4, which may account for the observed increases in their transactivation potential and reprogramming efficiency. Previously, Esch *et al*. demonstrated that the N-terminal short α-helix of the linker segment containing the E208 and E212 residues regulates the reprogramming efficiency of Oct4 by acting as a PPI interface with the key epigenetic players (12). Consequently, the moderately increased DNA binding affinity and favourable interactions with other pluripotency regulators may collectively contribute to the enhanced reprogramming efficiency of the E208A mutant. In contrast, the H-bond interaction mediated by E212 is not directly involved in the regulation of DNA binding and PPI during reprogramming. Taken together, our results demonstrate that in addition to acting as a PPI interface, the linker segment delicately regulates Oct4–DNA recognition via interaction with the RK-motif. Based on this evidence, we identified gain-of-function Oct4 mutants with enhanced DNA binding affinity and a nearly three-fold improvement of reprogramming efficiency.

**Figure 5.**
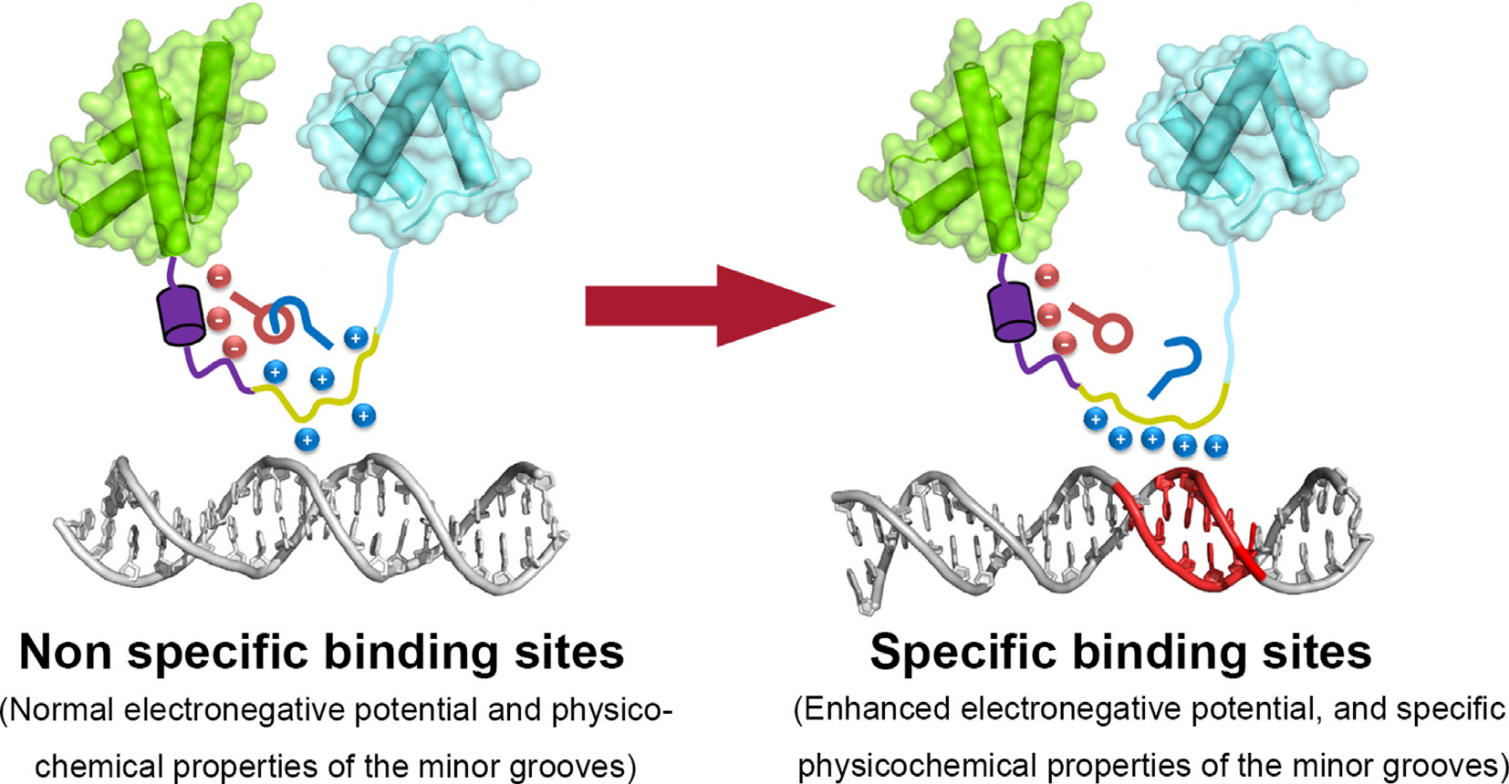
A model for the role of the interaction between the RK motif (yellow) and linker segment (purple) in specific DNA recognition by Oct4. The POUs subdomain is coloured green, and the POUh subdomain, except the RK-motif, is coloured cyan. The enhanced minor groove electrostatic potential at the narrowed minor groove is highlighted in red.

Based on the aforementioned results, we propose the following model for the functional interplay of the RK-motif and linker segment in Oct4–DNA binding (Figure 5). The dynamic and solvent-accessible RK-motif is essential to Oct4–DNA recognition through the perception of the enhanced electronegative potential and other important physicochemical properties, such as the specific hydration and hydrophobic environment in the narrowed minor groove. The H-bond interactions between the negatively charged glutamic acid residues in the linker segment and RK-motif may help to prevent the non-specific interactions of RK-motif with the random DNA sequences in genome. Upon associating with potential Oct4 binding sites containing A-tracts, the attractions from the enhanced minor groove electronegative potential may acts as a counterbalance to the H-bond interactions from the linker segment and help the release of the RK-motif for minor groove recognition. The similar situations were observed for other transcription factors, such as the ultrabithorax (Ubx) protein, in which the intramolecular interactions of disordered segments (I1, I2 and R region) and homeodomain constraint the non-specific interactions of homeodomain and thus increase its DNA binding specificity. (59,60) Moreover, the post-translational modifications (PTM) of the disordered region can modulate the DNA binding activities by intramolecular interactions with the binding determinants in some protein–DNA complexes. (61,62) It is thus speculated that the PTM in the linker segment of Oct4, such as the phosphorylation of serine or threonine, may affect its interplay with the RK-motif and thus the reprogramming efficiency. Correspondingly, albeit no data has been reported now, the potential PTMs or PPI with other regulatory factors of the solvent accessible RK-motif might achieve the similar effects.

Given the highly conserved nature of the RK motif and the recurrent emergence of negatively charged or polar residues in the linker segment of POU proteins (Supplementary Figures S1 and S2), the proposed regulatory mechanism may be universally adopted by other POU family members. Furthermore, the distinct number of negatively charged or polar residues and their diverse distributions along the linker segment may lead to the differential regulatory outputs and DNA binding properties of each POU protein. A previous study has found that the replacement of a glutamic acid residue with lysine in the linker segment of Oct1 results in a 2.5-fold reduction in DNA binding affinity (63). Our proposed model may therefore provide a plausible explanation for the functional specificity of different POU proteins. Recent computational and NMR spectroscopy studies indicate that the sliding, global jumping and intersegment transfer mechanisms are employed by Oct1 to facilitate the search for specific targets (64–67). The cooperative actions of the two globular subdomains of Oct1 evidently improve the search efficiency. In light of our current findings, further study is warranted to systematically compare the contributions of the RK-motif and its crosstalk with the linker segment in each of the relatively macroscopic mechanisms of Oct4. The gain-of-function Oct4 mutants identified here will be invaluable tools to manipulate the Oct4-related biological events with molecular precision and help to elucidate its role in pluripotency control.

## SUPPLEMENTARY DATA

Supplementary Data are available at NAR Online.

SUPPLEMENTARY DATA
