# Supplementary material for: Functional interplay between the RK motif and linker segment dictates Oct4–DNA recognition
Source: Nucleic Acids Res. 2015 Apr 13;43(9):4381–92. doi: 10.1093/nar/gkv323 (PMC4482079; doi:10.1093/nar/gkv323)
Supplement: SUPPLEMENTARY DATA [file supp_43_9_4381__index.html]

Functional interplay between the RK motif and linker segment dictates Oct4–DNA recognition — SUPPLEMENTARY DATA 

# Functional interplay between the RK motif and linker segment dictates Oct4–DNA recognition

## SUPPLEMENTARY DATA

**Files in this Data Supplement:**

- SUPPLEMENTARY DATA
