# Supplementary material for: Functional interplay between the RK motif and linker segment dictates Oct4–DNA recognition
Source: Nucleic Acids Res. 2015 Apr 13;43(9):4381–92. doi: 10.1093/nar/gkv323 (PMC4482079; doi:10.1093/nar/gkv323)
Supplement: SUPPLEMENTARY DATA [file supp_gkv323_nar-00191-z-2015-File007.doc]

**SI Appendix for:**

**Functional Interplay Between the RK motif and Linker Segment Dictates Oct4-DNA Recognition**

Xiangqian Kong^a,1^, Jian Liu^b,1^, Lianchun Li^a^, Liyan Yue^a^, Lihong Zhang^b^, Hualiang Jiang^a^, Xin Xie^b,2^, Cheng Luo^a,2^

^a^Drug Discovery and Design Center, State Key Laboratory of Drug Research, Shanghai Institute of Materia Medica, Chinese Academy of Sciences, Shanghai 201203, China; ^b^Chinese Academy of Sciences Key Laboratory of Receptor Research, National Center for Drug Screening, Shanghai Institute of Materia Medica, Chinese Academy of Sciences, Shanghai 201203, China.

^1^These authors contributed equally to this work.

^2^To whom correspondence should be addressed. E-mail: [xxie@simm.ac.cn](mailto:xxie@simm.ac.cn) or [cluo@simm.ac.cn](mailto:cluo@simm.ac.cn)

**SI Appendix Figure S1.**


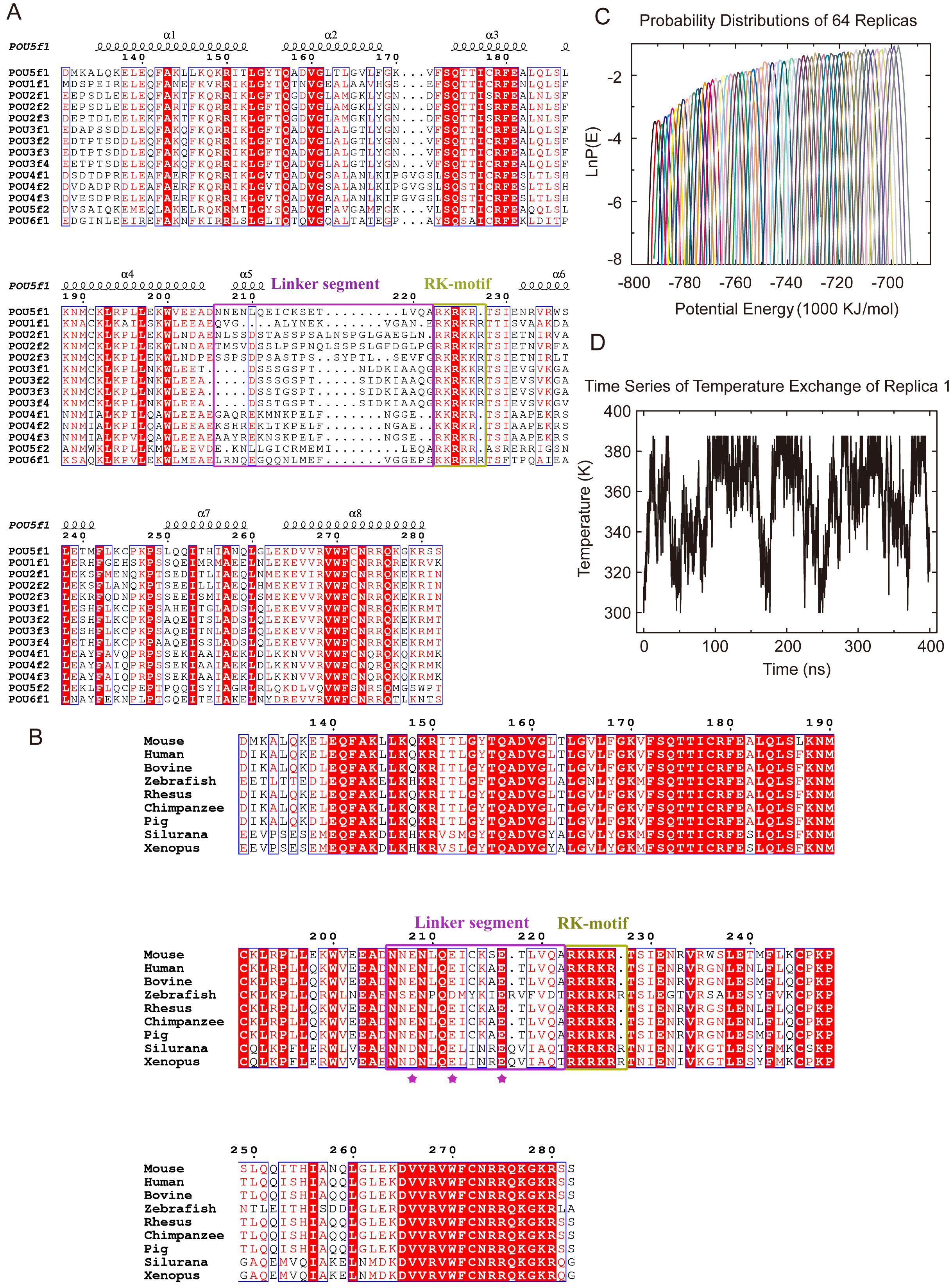


(A) Sequence alignment of POU family members. Both the RK-motif and linker segment are highlighted. (B) Sequence alignment of Oct4 from various species. The conserved glutamic acid residues in the linker segment are highlighted. (C) The canonical probability distributions between all neighboring pairs of temperatures used in the REMD simulations. (D) The time series of temperature exchange of replica 1.

**SI Appendix Figure S2.**

**
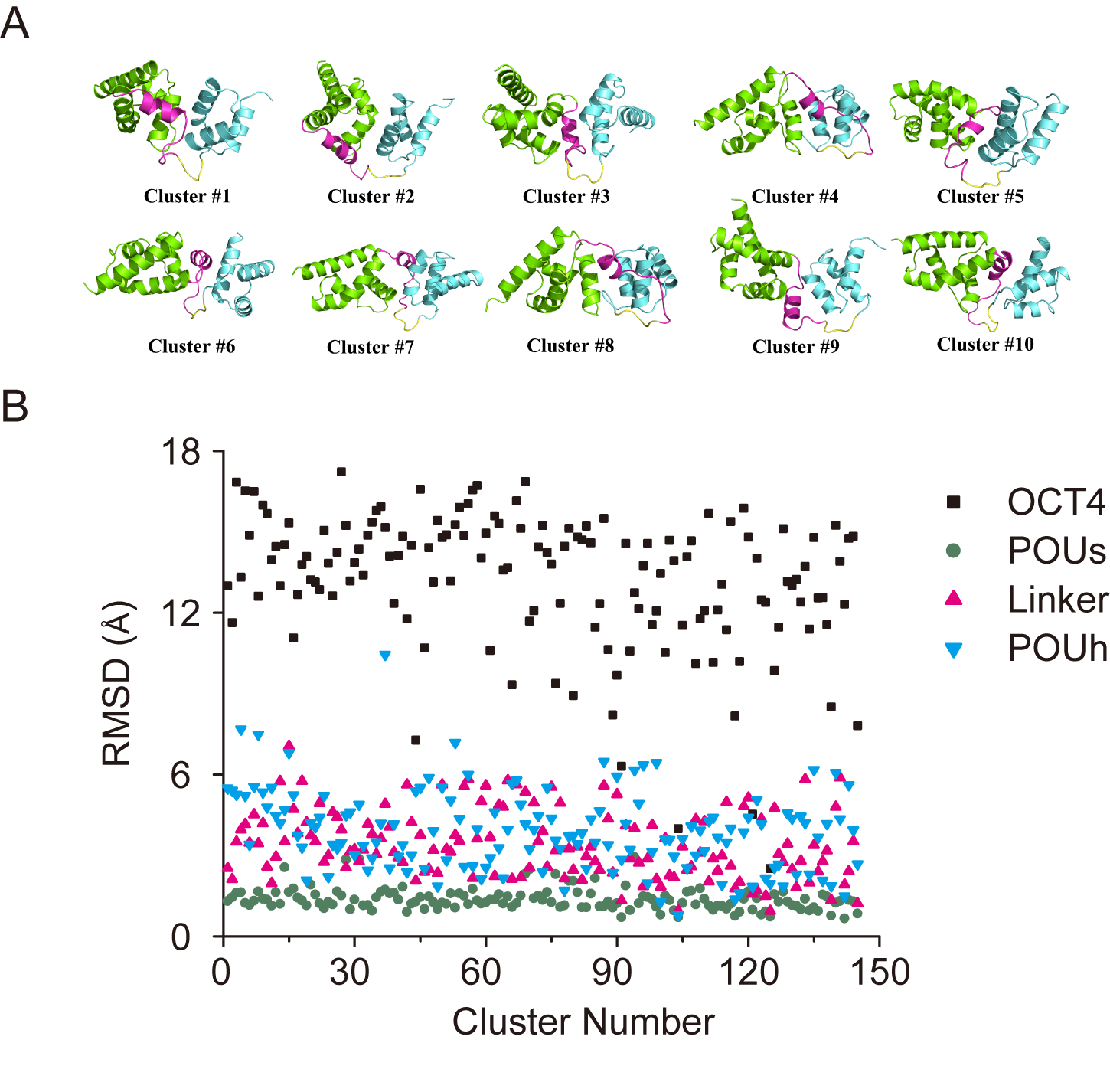
**

(A) The representative conformations of the apo-state OCT4 in the top 10 clusters obtained in the REMD simulations. (B) The root-mean-square deviations (RMSD) for the backbone atoms of POUs subdomain, POUh subdomain, linker segment and OCT4 from their initial positions in each cluster.

**SI Appendix Figure S3.**

**
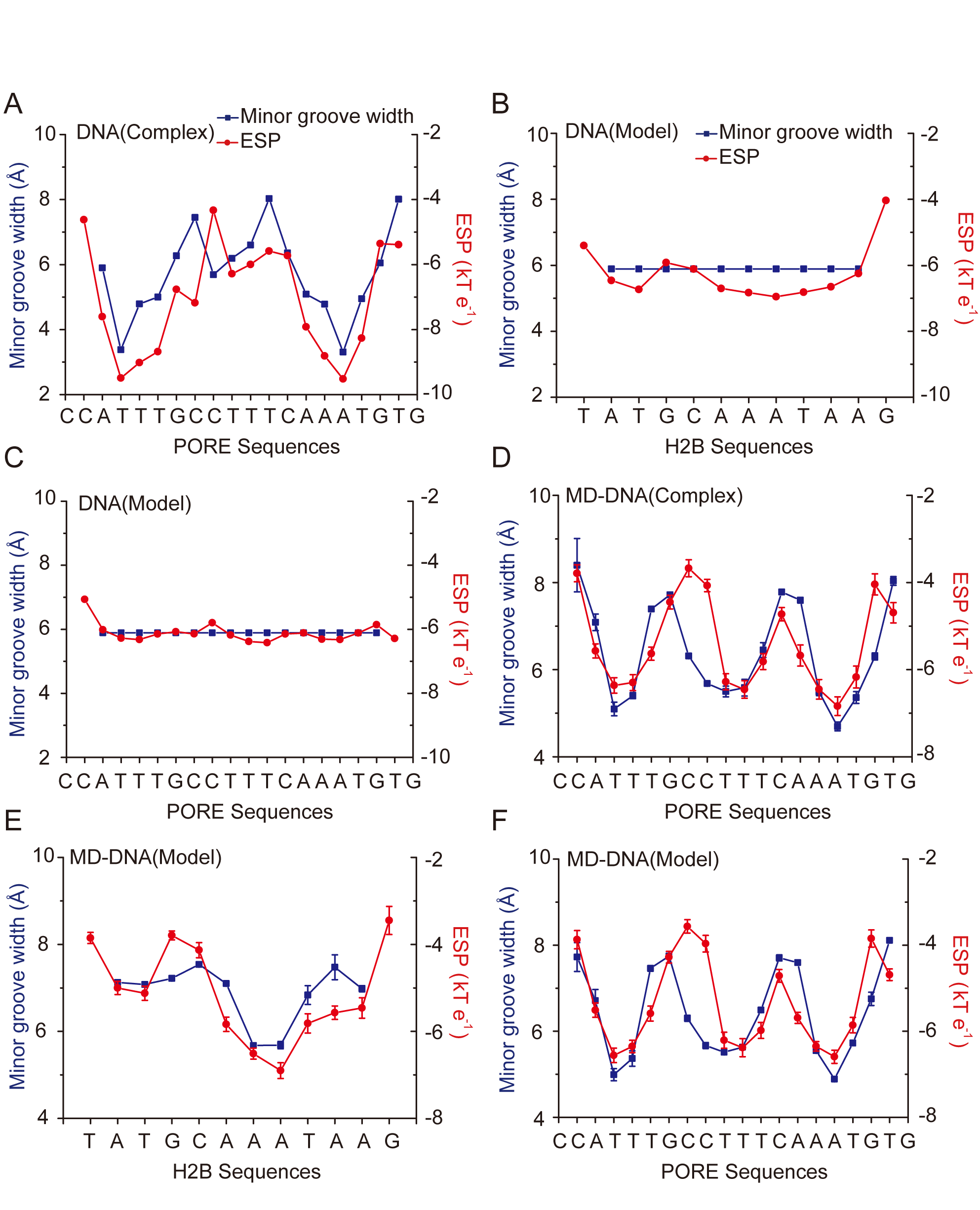
**

(A, B, C) The minor groove width and electrostatic potential of the PORE motif in the modeled Oct4-DNA complex (A), 3DNA generated standard B-form DNA for H2B (B) and PORE sequences (C). (D, E, F) The minor groove width and electrostatic potential in the MD simulation with the initial DNA conformation from the modeled Oct4-PORE complex (D), 3DNA generated standard B-form DNA for H2B (E) and PORE motifs (F). The data (Means ± SD) are obtained from three independent MD simulations.

**SI Appendix Figure S4.Western blot analysis of the protein levels of the exogenous WT and mutantOct4.**


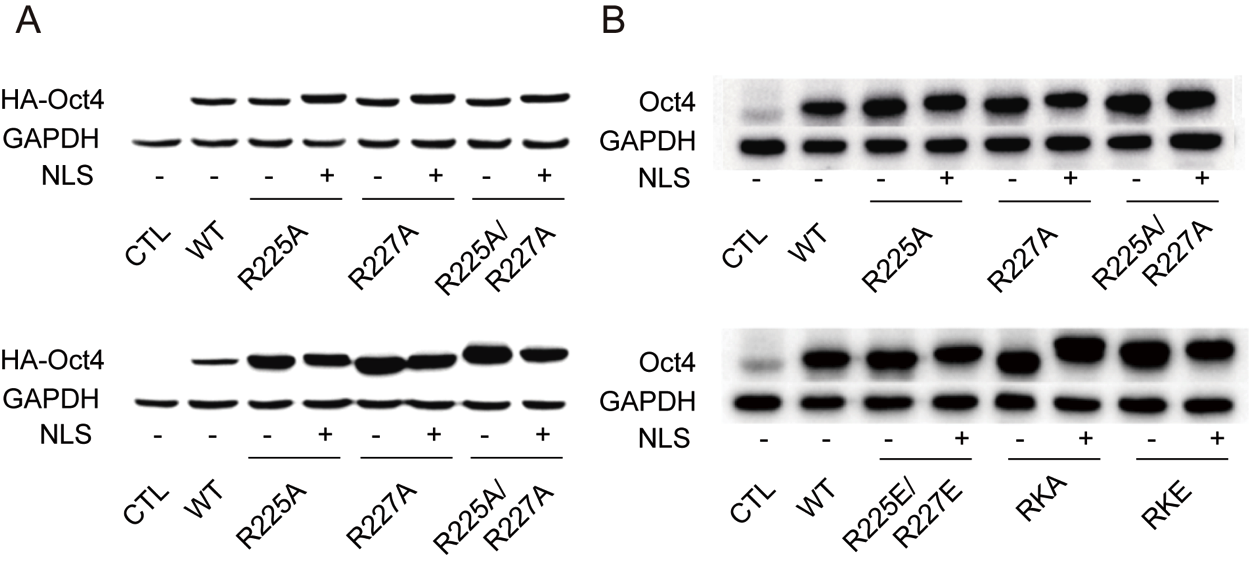


(A) and (B) Representative western blots of various forms of Oct4 corresponding to Figure 3D (A) and Figure 3E (B).

**SI Appendix Figure S5.The characterization of iPSCs.**

**
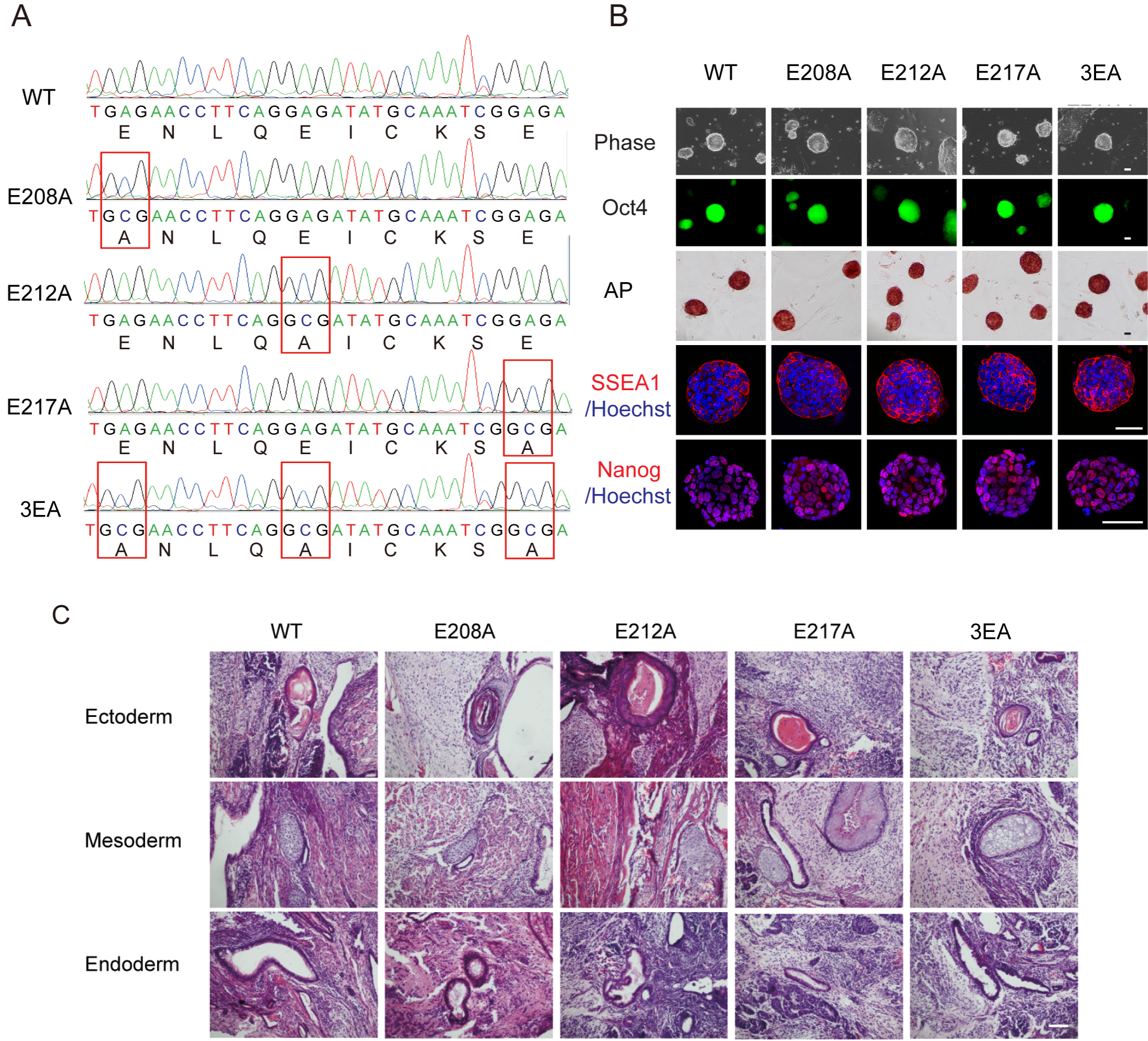
**

(A) The integration of Oct4 mutants in the genome of iPSCs was detected by sequencing. (B) Morphology, GFP expression, AP staining, immunofluorescent staining of SSEA-1 and Nanog in the corresponding iPSCs in Figure4E. Hoechst was used to mark nuclei. Scale bars, 50μm. (C) H&E staining of teratomas generated with corresponding iPSCs in Figure 4E. Typical structures of the three embryonic germ layers are shown. Scale bars, 50μm.

**SI Appendix Table S1. Acceptance ratios of replica exchange corresponding to pairs of neighboring temperatures**

| **Pair of Temperatures** | **Acceptance Ratio** | **Pair of Temperatures** | **Acceptance Ratio** |
| --- | --- | --- | --- |
| 300.00K <-> 301.25K | 0.24 | 342.15K <-> 343.54K | 0.26 |
| 301.25K <-> 302.50K | 0.24 | 343.54K <-> 344.93K | 0.26 |
| 302.50K <-> 303.76K | 0.23 | 344.93K <-> 346.33K | 0.26 |
| 303.76K <-> 305.02K | 0.24 | 346.33K <-> 347.74K | 0.26 |
| 305.02K <-> 306.28K | 0.24 | 347.74K <-> 349.14K | 0.26 |
| 306.28K <-> 307.56K | 0.24 | 349.14K <-> 350.56K | 0.26 |
| 307.56K <-> 308.83K | 0.24 | 350.56K <-> 351.98K | 0.26 |
| 308.83K <-> 310.11K | 0.24 | 351.98K <-> 353.40K | 0.26 |
| 310.11K <-> 311.39K | 0.25 | 353.40K <-> 354.83K | 0.26 |
| 311.39K <-> 312.68K | 0.24 | 354.83K <-> 356.26K | 0.26 |
| 312.68K <-> 313.97K | 0.24 | 356.26K <-> 357.67K | 0.27 |
| 313.97K <-> 315.26K | 0.25 | 357.67K <-> 359.11K | 0.26 |
| 315.26K <-> 316.56K | 0.25 | 359.11K <-> 360.56K | 0.26 |
| 316.56K <-> 317.87K | 0.24 | 360.56K <-> 362.01K | 0.26 |
| 317.87K <-> 319.17K | 0.25 | 362.01K <-> 363.46K | 0.26 |
| 319.17K <-> 320.52K | 0.23 | 363.46K <-> 364.92K | 0.26 |
| 320.52K <-> 321.83K | 0.25 | 364.92K <-> 366.39K | 0.26 |
| 321.83K <-> 323.15K | 0.24 | 366.39K <-> 367.86K | 0.26 |
| 323.15K <-> 324.48K | 0.25 | 367.86K <-> 369.33K | 0.27 |
| 324.48K <-> 325.81K | 0.25 | 369.33K <-> 370.82K | 0.26 |
| 325.81K <-> 327.15K | 0.25 | 370.82K <-> 372.30K | 0.27 |
| 327.15K <-> 328.48K | 0.25 | 372.30K <-> 373.79K | 0.27 |
| 328.48K <-> 329.84K | 0.25 | 373.79K <-> 375.29K | 0.27 |
| 329.84K <-> 331.19K | 0.25 | 375.29K <-> 376.79K | 0.27 |
| 331.19K <-> 332.55K | 0.25 | 376.79K <-> 378.30K | 0.27 |
| 332.55K <-> 333.90K | 0.25 | 378.30K <-> 379.81K | 0.27 |
| 333.90K <-> 335.27K | 0.25 | 379.81K <-> 381.33K | 0.27 |
| 335.27K <-> 336.63K | 0.25 | 381.33K <-> 382.85K | 0.27 |
| 336.63K <-> 338.00K | 0.25 | 382.85K <-> 384.38K | 0.27 |
| 338.00K <-> 339.38K | 0.25 | 384.38K <-> 385.91K | 0.27 |
| 339.38K <-> 340.76K | 0.26 | 385.91K <-> 387.45K | 0.27 |
| 340.76K <-> 342.15K | 0.25 |  |  |

**SI Appendix Table S2. Primers used to generate Oct4 mutations**

| **Mutation** | **primer** | |
| --- | --- | --- |
| R223A | sense | CCCTGGTGCAGGCCGCGAAGAGAAAGCGAA |
|  | anti-sense | TTCGCTTTCTCTTCGCGGCCTGCACCAGGG |
| K224A | sense | CTGGTGCAGGCCCGGGCGAGAAAGCGAACTAGC |
|  | anti-sense | GCTAGTTCGCTTTCTCGCCCGGGCCTGCACCAG |
| R225A | sense | GGTGCAGGCCCGGAAGGCAAAGCGAACTAGCATTG |
|  | anti-sense | CAATGCTAGTTCGCTTTGCCTTCCGGGCCTGCACC |
| K226A | sense | GCAGGCCCGGAAGAGAGCGCGAACTAGCATTGAG |
|  | anti-sense | CTCAATGCTAGTTCGCGCTCTCTTCCGGGCCTGC |
| R227A | sense | GGCCCGGAAGAGAAAGGCAACTAGCATTGAGAACC |
|  | anti-sense | GGTTCTCAATGCTAGTTGCCTTTCTCTTCCGGGCC |
| R225A/R227A | sense | GGCCCGGAAGGCAAAGGCAACTAGCATTGAGAAC |
|  | anti-sense | GTTCTCAATGCTAGTTGCCTTTGCCTTCCGGGCC |
| R225E/R227E | sense | GACCCTGGTGCAGGCCCGGAAGGAGAAGGAGACTAGCATTGAGAACCGTGTGAG |
|  | anti-sense | CTCACACGGTTCTCAATGCTAGTCTCCTTCTCCTTCCGGGCCTGCACCAGGGTC |
| RKRKR(223~227)AAAAA  (RKA) | sense | CAAATCGGAGACCCTGGTGCAGGCCGCGGCGGCAGCGGCAACTAGCATTGAGAACCGTGTGAGG |
|  | anti-sense | CCTCACACGGTTCTCAATGCTAGTTGCCGCTGCCGCCGCGGCCTGCACCAGGGTCTCCGATTTG |
| RKRKR(223~227)EEEEE  (RKE) | sense | CGGAGACCCTGGTGCAGGCCGAGGAGGAGGAGGAGACTAGCATTGAGAACCGTGTG |
|  | anti-sense | CACACGGTTCTCAATGCTAGTCTCCTCCTCCTCCTCGGCCTGCACCAGGGTCTCCG |
| E208A | sense | GGAAGCCGACAACAATGCGAACCTTCAGGAGATATG |
|  | anti-sense | CATATCTCCTGAAGGTTCGCATTGTTGTCGGCTTCC |
| E212A | sense | CAATGAGAACCTTCAGGCGATATGCAAATCGGAGAC |
|  | anti-sense | GTCTCCGATTTGCATATCGCCTGAAGGTTCTCATTG |
| E217A | sense | GAGATATGCAAATCGGCGACCCTGGTGCAGGC |
|  | anti-sense | GCCTGCACCAGGGTCGCCGATTTGCATATCTC |
| E208A/E212A/E217A | sense | GAGGAAGCCGACAACAATGCGAACCTTCAGGCGATATGCAAATCGGCGACC |
|  | anti-sense | GGTCGCCGATTTGCATATCGCCTGAAGGTTCGCATTGTTGTCGGCTTCCTC |
| pMXs+NLS | sense | CGGGATCCATGGCTGGACACCTGGCT |
|  | anti-sense | CCGCTCGAGTCATCGTCGCTTCTTTCTCCGGTTTGAATGCATGGGAGAGCCCAGA |
| pcDNA3.1-HA+NLS | sense | GGAATTCTCATGGCTGGACACCTGGCT |
|  | anti-sense | CCGCTCGAGTCATCGTCGCTTCTTTCTCCGGTTTGAATGCATGGGAGAGCCCAGA |

**SI Appendix Table S3.Primers used in ChIP-PCR analysis**

| **Locus** | **Forward primer** | **Reverse primer** |
| --- | --- | --- |
| *Oct4*promoter | AGGGAGGTTGAGAGTTCT | AGGGCTAGGACGAGAGG |
| *Sox2*promoter | TTTATTCAGTTCCCAGTCCAA | TTATTCCTATGTGTGAGCAAGA |
| *Jarid2*promoter | TGTTTGGTTTCATTTCCC | CTATAATGTGCCCCACAA |
| *Negative Control* | AGCATGTGTTCTTCTTACCA | GTTAGTTCATATTATTGTTCCACCTATA |
| *GAPDH* CDS | CCTTCATTGACCTCAACTACA | TAGACTCCACGACATACTCA |

**SI Materials and Methods**

**Preparation of the simulation system**

To construct the molecular model for Oct4-PORE complex, the loop-search method included in the Biopolymer module of Sybyl software package (Tripos, St. Louis, MO.) was firstly used to generate the coordinates for the residues invisible in the linker segment (residue 217-219) of Oct4-PORE structure (PDB entry: 3L1P). Then the coordinates of the disordered sidechains were built and their orientations were optimized to avoid the steric clash with other residues. The resulting Oct4-PORE complex was minimized by using the AMBER99SB force field with the following parameters: a distance-dependent dielectric function, nonbonded cutoff of 8 Å, Amber charges for the protein and DNA. The structure was minimized by the simplex method, followed by the Powell method to an energy gradient<0.05 kcal/(mol·Å). All procedures were performed using the Sybyl software package (Tripos, St. Louis, MO).

The initial configuration of the Oct4-H2B complex was modeled based on the crystal structure of Oct1-H2B complex (PDB entry: 1OCT) and the modeled Oct4-PORE complex. The ClustalW2 program was employed to generate the pairwise sequence alignment of the POU domains between Oct1 and Oct4. Then a preliminary homology model of the POU domain of Oct4 in the DNA-binding state was built by the MODELER program in Discovery Studio 2.6 (Accelrys Software Inc.), using Oct1-H2B complex as the template. As the linker segment is invisible in the Oct1-H2B complex, the linker structure in Oct4-PORE complex was extracted and merged to Oct4-H2B complex after structural alignment. The resulting protein-DNA complex was minimized as aforementioned to eliminate the possible intramolecular clashes.

The 3DNA program was employed to build the standard B-form DNA models with the sequences of both H2B and PORE as inputs.

**Molecular dynamics simulation**

Four systems were set up with the initial DNA conformation from Oct4-H2B complex, Oct4-PORE complex, and 3DNA generated models for H2B and PORE, respectively. All of the MD simulations were carried out using the GROMACS 4.5.3 package with constant temperature and pressure and periodic boundary conditions. The Amber99SB force field with parmbsc0 modifications and TIP3P model for water molecules were used. During MD simulations, all bonds involving hydrogen atoms were constrained with the LINCS algorithm, and the integration step of 2 fs was used. Electrostatic interactions were calculated using the particle-mesh Ewald method. The nonbonded cutoff was set to 10.0 Å, and the nonbonded pairs were updated every 10 steps. The temperature was kept constant at 300 K by coupling the protein, water, and ions separately in a thermal bath using the modified Berendsen thermostat with a coupling time of 0.1 ps. A constant pressure of 1.0 bar was applied isotropically to the systems, with a coupling constant of 1.0 ps. Firstly, the systems were subjected to energy minimizations using the steepest-descents algorithm to remove unfavorable contacts. After minimization of the whole system, the first 500-ps MD simulation was carried out to heat the solvent molecules and ions to 300 K with the DNA fixed. After that, the second 500-ps MD simulations were performed to heat all of the atoms in the system to 300 K with the DNA backbone fixed. Third, with the whole system relaxed and the equilibration was completed after 1 ns MD simulation. Finally, three independent 200-ns MD simulations were performed for each system, with coordinates saved every 10ps during the entire process.

**Replica-exchange molecular dynamics simulation**

The configuration of Oct4 in the protein-PORE complex was employed as the initial conformation in the simulations. The protein was solvated into a rectangular box with a 10 Å buffer distance between the solvent box wall and the nearest solute atoms, and the counterions were added to the system to neutralize the simulation system. Then the energy minimization procedure was performed on the system to eliminate unfavorable contacts. The temperatures used in the REMD simulations were chosen based on the criteria that a good overlap between energy distributions and significant acceptance ratios should be simultaneously achieved. The temperature range was chosen between 300 and 390 K, with 1-2 K spacing resulting in 64 replicas and 20% anticipated exchange acceptance ratio by using the T-REMD server. Before running the REMD simulations, the system was replicated and each replica was slowly heated to the defined target temperature with protein fixed in a 5-ns MD simulations. Then the density of water molecules around the protein was equilibrium by another 10-ns MD simulations for each replica in the constant temperature and pressure (NPT) ensemble. Finally, the temperature exchanges between adjacent replicas were initiated and each replica was simulated in the NPT ensemble for 400-ns in the REMD simulations. The time interval between the exchange attempts was set to 2ps. All of the MD and REMD simulations were carried out using the GROMACS 4.5.3 package with periodic boundary conditions. The AMBER99SB force field and TIP3P model for water molecules were used in both the preceding preparatory steps and REMD simulations. The other parameters were the same as that in the conventional MD simulations.

**Calculation of minor groove width and electrostatic potential**

The groove geometry and electrostatic potential were calculated as previously described. The Curve+ program was employed to analyze the minor groove width of DNA as a function of base sequences. Electrostatic potentials were calculated by using the DelPhi program to solve the non-linear Poisson-Boltzman equation at physiologic ionic strength (0.145 M). The electrostatic potentials at geometric midpoints between the O4’ atoms of nucleotide i+1 on the 5’ strand and nucleotide i-1 on the 3’ strand were represented as the minor groove potentials. Partial charges and radii were obtained from Amber force field. The interior of the macromolecules (probed by a 1.4 Å sphere) was assigned a dielectric constant of ε=2 while the exterior solvent was assigned a value of 80. The grid size was set so that the final grid spacing was <0.5 Å after five focusing steps from 10% to 90% space filling.

**Plasmid construction and Site-specific mutagenesis of Oct4**

Site-specific Oct4 mutant in the RKRKR motif or linker region were constructed with KOD-Plus-kit (TOYOBO) according to the manufacturer's protocol. In brief, pMXs-Oct4, pcDNA3.1-HA-Oct4 or pEF-HA-Oct4 plasmids were used as templates for PCR with primers carrying corresponding mutations (SI Appendix Table S2). After PCR, the methylated parental DNA templates were digested with 20U DpnI (TOYOBO, DPN-101) at 37°C for 2h. The remaining DNA was then transformed into competent *Escherichia coli* TOP10 cells, in which nicked DNA was repaired. Corresponding mutations were verified by sequencing. For adding exogenous NLS, mutants of OCT4 were used as template for PCR with corresponding NLS primers and cloned into corresponding vector.

For protein expression, DNA sequence encoding POU domain of Oct4 was cloned into a modified pET28a plasmid, which encodes an N-terminal His6-SUMO (small ubiquitin-related modifier) tag, by *Nde* I and *Xho*I restriction sites. Mutations were introduced by the QuickChange II site-directed mutagenesis kit (Stratagene, La Jolla, CA). All plasmids were confirmed by sequencing.

**Protein expression and purification**

To express wide-type (WT) POU domain, *E.coli* BL21 (DE3) cells were transformed with the expression plasmid and cultured in L.B. (Luria-Bertani) culture medium with 50μg/ml kanamycin at 37°C with shaking. When OD600 reach 0.6, the culture were transferred to 16°C and induced with 0.4 mM Isopropyl β-D-1-thiogalactopyranoside (IPTG) overnight. Cells were collected by centrifugation and the pellets were re-suspended in lysis buffer (50mM Tris-HCl, pH 7.4, 200mM NaCl, 20mM imidazole, 10mM β-mercaptoethanol, 20μg/ml DNAse I and 4mM MgCl_2_), then disrupted by sonication. The debris was removed by centrifugation (39000g for 30min at 4°C) and the clarified supernatant was purified by HisTrap FF and HiTrap SP FF column (GE Healthcare). The purified protein was concentrated by ultrafiltration, then flash frozen in liquid nitrogen and stored at -80°C. Expression and purification of the mutants were performed with the same methods as the WT.

**Electrophoretic Mobility Shift Assay (****EMSA)**

Briefly, 20μl of the mixture of the DNA probe (0.25 μM) containing single octamer motif, purified POU proteins (0.7μM for E208A, E212A, E217A and E208A/E212A/E217A and 1μM for other mutants) in binding buffer (10mM Tris-HCl, pH7.8, 50mM KCl, 0.5mM EDTA, 1mM DTT, 3% glycerol) were incubated at room temperature for 1h. Then the samples were loaded onto a native polyacrylamide gel (5%) and electrophoresed in 0.5×TBE buffer (50mM Tris, 41.5mM borate, pH 8.0) at room temperature. The gel was stained in GelRed nucleic acid staining solution (Biotium) for 10 min, and then the DNA bands were visualized. The intensity of the DNA bands was quantified by the GIS 1D analysis software (Tanon). The DNA sequences used in the assay are listed below, and the Oct4 binding sites are highlighted.

5’-CTAGAAGAATCGCTT**ATGCAAAT**AAGGTGAAGATCTAG-3’

5’-CTAGATCTTCACCTT**ATTTGCAT**AAGCGATTCTTCTAG-3’

**Cell culture and Derivation of MEFs**

HEK293T cells and Platinum E (plat-E) retroviral packaging cells were maintained in DMEM medium supplemented with 10% (vol/vol) FBS, 100units/ml penicillin and 100μg/ml streptomycin. OG2 mice, which carry the Rosa26-lacZ allele and a transgenic Oct4 promoter driven GFP expression, were mated with C57 mice. MEFs were isolated from E12.5embryos. Gonads and internal organs were removed before MEF isolation. MEFs were grown in DMEM supplemented with 10% (vol/vol) FBS, 2mM L-glutamax, 0.1mM nonessential amino acids (NEAA), 100 units/ml penicillin and 100 μg/ml streptomycin. Isolated MEFs in passage 1 were used for further experiments.

## Luciferase reporter assay

HEK293T cells were seeded onto 96-well plates at a density of 25,000 cell/well. Next day, constructs encoding HA-Oct4 (WT or mutant) (25 ng/well), firefly luciferase reporter under the control of TK promoter and 6W enhancer (25 ng/well), and the control Renilla luciferase reporter (pRL-TK，2.5 ng/well) were transfected into the cells using Fugene transfection reagent (Life Technology). Firefly and renilla luciferase activities were measured 24 hrs later with the Dual-Glo Luciferase Assay System (Promega) using an EnVision multilabel plate reader (Perkin Elmer). The firefly luciferase activity was normalized to the Renilla luciferase activity in the same sample.

**Mouse iPSC generation**

Retrovirus were produced by transfection of plat-Ecells with pMXs retroviral vectors containing the coding sequences of mouse Oct4 (WT or mutant), Sox2 and Klf4. MEFs carrying an Oct4-GFP reporter were seeded at a density of 150,000 cells per well in six-well plate 18 hrs before infection. Virus containing supernatants, supplemented with 4 μg/ml polybrene, were added onto the plates of MEF cultures and spined at 2,500 rpm for 90 min to ensure their infection. Medium was changed immediately after virus transduction. Two days post virus infection, MEFs were digested into single cells and reseeded at a density of 10,000 cells per well on 24-well plates pre-seeded with irradiated MEF feeders, supplemented with mES medium (DMEM supplemented with 15% FBS, 2 mM L-glutamax, 0.1 mM NEAA, 0.1 mM β-mercaptoethanol 1,000 U/ml LIF, 100 units/ml penicillin and 100 μg/ml streptomycin). At day 6, culture medium was replaced with KSR medium (knockout-DMEM supplemented with 15% knockout serum replacement, 2 mM L-glutamax, 0.1 mM nonessential amino acids (NEAA), 0.1 mM β-mercaptoethanol, 1,000 U/ml LIF, 100 units/ml penicillin and 100 μg/ml streptomycin). Images of representative wells were taken and GFP^+^ colonies were counted using an Olympus IX71 inverted fluorescent microscope and Image Pro Plus software at day 16 post infection.

**Alkaline phosphatase (AP) and immunofluorescent staining**

## For AP staining, iPSCs were fixed with 4% PFA in PBS for 30s, rinsed once with PBS and detection was performed using a leukocyte AP kit (Sigma, catalog No 85L3R) according to the manufacturer's protocol. For immunofluorescent staining, cells were fixed with 4% PFA and incubated with anti-SSEA-1 (Santa Cruz, sc-21702) or anti-Nanog (Millipore, AB5731) antibody, followed by secondary antibody conjugated to Alexa Fluor 555. Nuclei were counterstained with Hoechst 33342 (Sigma-Aldrich, H2261). Images were taken with Olympus IX51 inverted fluorescent microscope or Olympus FV10i confocal microscope.

To analyze the subcellular localization ofOct4, HEK293T cells were transfected with pcDNA3.1-HA-Oct4 (WT or mutant with or without additional NLS). Twenty-four hours later, cells were fixed, permeablized and the exogenous Oct4 expression was probed with TRITC-conjugated anti-HA antibody (Sigma-Aldrich, H9037, 1:500). Nuclei were counterstained with Hoechst. Images were taken with an Olympus FV10i confocal microscope. The percentage of Oct4 localized in the nuclei was analyzed with Image J software.

**Western blot**

Cells were lysed, sonicated and boiled at 95-100 °C for 5 min in sample buffer (50 mM Tris-HCl, 2% w/v SDS, 10% glycerol, 1% β-mercaptoethanol, 0.01% bromophenyl blue (pH 6.8)). Cell lysates were separated on SDS-PAGE and transferred to polyvinylidene difluoride membranes. The membranes were first incubated with blocking buffer (TBS with 0.1% Tween 20, 5% nonfat milk) for 1 hrs at room temperature and then incubated overnight at 4 °C in buffer containing rabbit anti-GAPDH (Cell Signaling, 2118s), rabbit anti-HA (Santa Cruz, sc-805) or mouse anti-Oct4 (Millipore, MAB4419). The membranes were washed thrice and incubated with goat anti-rabbit IgG HRP (Abmart, M21002L) or goat anti-mouse IgG HRP (Abmart, M21001L) for 1 hrs. After washing, immunostaining was visualized using Amersham ECL Plus Western Blotting detection reagents (GE RPN2232).

**Teratoma formation**

About 1 × 10^6^ iPSCs were suspended in 200 μL mES medium and injected into the thigh muscle of NOD-SCID mice. The animals were checked 2–3 times per week. Four weeks after injection, teratomas were harvested, fixed overnight with 4% PFA, embedded in paraffin, and sectioned. Sections of the teratomas were stained with hematoxylin and eosin and analyzed histologically.

**ChIP assay and quantitative PCR**

E14 mESCs were transfected with pEF-HA-Oct4 (WTor mutant) using GeneExpresso™ Max transfection reagent (Excellgen, EG-1086). 48 hrs later 3×10^7^ cells were fixed with 1% formaldehyde for 10 min at room temperature and then washed with 125 mM glycine for 5 min and then with cold PBS. Cells were then lysed in ChIP buffer A (5mM PIPES (pH 8.0), 85mM KCl, 0.5% NP-40 and protease inhibitor cocktail) for 10 min at 4 °C. Samples were centrifuged at 1,200 g for 5 min at 4 °C. Pellets were resuspended in ChIP buffer B (1% SDS, 50 mM Tris-HCl (pH 8.0), 10 mM EDTA and protease inhibitor cocktail) for 10 min at 4 °C and then sheared by sonication to obtain an average DNA fragment size of 500 base pairs. Samples were centrifuged at 13,000 g for 15 min at 4 °C, and the supernatants were diluted (1:1) with ChIP IP buffer (0.01% SDS, 1% Triton X-100, 1.2 mM EDTA, 16.7 mM Tris-HCl (pH 8.0), 167 mM NaCl and protease inhibitor cocktail). Antibodies (anti-Oct4 (abcam19857) or control IgG (Millipore 12-370)) were incubated with sheared chromatin overnight at 4 °C. Next day, antibodies were coupled to protein G beads (Milipore 16-201) for 3 hrs at 4 °C and beads were washed with low-salt wash buffer (0.1% SDS, 1% Triton X-100, 2 mM EDTA, 20 mM Tris-HCl (pH 8.0) and 150 mM NaCl), high-salt wash buffer (0.1% SDS, 1% Triton X-100, 2 mM EDTA, 20 mM Tris-HCl (pH 8.0) and 300 mM NaCl), LiCl wash buffer (0.25 M LiCl, 1% NP-40, 1% deoxycholate, 1 mM EDTA and 10 mM Tris-HCl (pH 8.1)) and TE buffer (10 mM Tris-HCl and 1 mM EDTA (pH 8.0)). Chromatin was eluted for 30min at room temperature in elution buffer (1% SDS and 0.1M NaHCO3). Samples were incubated overnight at 65 °C to reverse the crosslinking. Next day, RNase was added and incubated at 37 °C for 1 hrs. Then proteinase K was added and incubated at 55 °C for 1 hrs. The DNA was phenol:chloroform extracted and ethanol precipitated. Samples were eluted in TE buffer and used for analysis. The sequences of all primers used are listed in SI Appendix Table S3.

**Statistic Analysis**

Values are reported as the Means ± SEM and analyzed using two-tailed Student’s t-test. P<0.05 was considered statistically significant.

**References**

1. Esch, D., Vahokoski, J., Groves, M.R., Pogenberg, V., Cojocaru, V., vom Bruch, H., Han, D., Drexler, H.C.A., Arauzo-Bravo, M.J., Ng, C.K.L. *et al.* (2013) A unique Oct4 interface is crucial for reprogramming to pluripotency. *Nat Cell Biol*, **15**, 295-301.

2. Klemm, J.D., Rould, M.A., Aurora, R., Herr, W. and Pabo, C.O. (1994) Crystal structure of the Oct-1 POU domain bound to an octamer site: DNA recognition with tethered DNA-binding modules. *Cell*, **77**, 21-32.

3. Larkin, M.A., Blackshields, G., Brown, N.P., Chenna, R., McGettigan, P.A., McWilliam, H., Valentin, F., Wallace, I.M., Wilm, A., Lopez, R. *et al.* (2007) Clustal W and Clustal X version 2.0. *Bioinformatics*, **23**, 2947-2948.

4. Lu, X.J. and Olson, W.K. (2003) 3DNA: a software package for the analysis, rebuilding and visualization of three-dimensional nucleic acid structures. *Nucleic Acids Res*, **31**, 5108-5121.

5. Van Der Spoel, D., Lindahl, E., Hess, B., Groenhof, G., Mark, A.E. and Berendsen, H.J. (2005) GROMACS: fast, flexible, and free. *J Comput Chem*, **26**, 1701-1718.

6. Perez, A., Marchan, I., Svozil, D., Sponer, J., Cheatham, T.E., 3rd, Laughton, C.A. and Orozco, M. (2007) Refinement of the AMBER force field for nucleic acids: improving the description of alpha/gamma conformers. *Biophys J*, **92**, 3817-3829.

7. Jorgensen, W.L., Chandrasekhar, J., Madura, J.D., Impey, R.W. and Klein, M.L. (1983) Comparison of Simple Potential Functions for Simulating Liquid Water. *J Chem Phys*, **79**, 926-935.

8. Hess, B., Bekker, H., Berendsen, H.J.C. and Fraaije, J.G.E.M. (1997) LINCS: A linear constraint solver for molecular simulations. *Journal of Computational Chemistry*, **18**, 1463-1472.

9. Darden, T., York, D. and Pedersen, L. (1993) Particle Mesh Ewald - an N.Log(N) Method for Ewald Sums in Large Systems. *J Chem Phys*, **98**, 10089-10092.

10. Berendsen, H.J.C., Postma, J.P.M., Vangunsteren, W.F., Dinola, A. and Haak, J.R. (1984) Molecular-Dynamics with Coupling to an External Bath. *J Chem Phys*, **81**, 3684-3690.

11. Sugita, Y. and Okamoto, Y. (1999) Replica-exchange molecular dynamics method for protein folding. *Chem Phys Lett*, **314**, 141-151.

12. Patriksson, A. and van der Spoel, D. (2008) A temperature predictor for parallel tempering simulations. *Phys Chem Chem Phys*, **10**, 2073-2077.

13. Cornell, W.D., Cieplak, P., Bayly, C.I., Gould, I.R., Merz, K.M., Ferguson, D.M., Spellmeyer, D.C., Fox, T., Caldwell, J.W. and Kollman, P.A. (1996) A second generation force field for the simulation of proteins, nucleic acids, and organic molecules (vol 117, pg 5179, 1995). *J Am Chem Soc*, **118**, 2309-2309.

14. Wang, J.M., Cieplak, P. and Kollman, P.A. (2000) How well does a restrained electrostatic potential (RESP) model perform in calculating conformational energies of organic and biological molecules? *Journal of Computational Chemistry*, **21**, 1049-1074.

15. Hornak, V., Abel, R., Okur, A., Strockbine, B., Roitberg, A. and Simmerling, C. (2006) Comparison of multiple amber force fields and development of improved protein backbone parameters. *Proteins*, **65**, 712-725.

16. Joshi, R., Passner, J.M., Rohs, R., Jain, R., Sosinsky, A., Crickmore, M.A., Jacob, V., Aggarwal, A.K., Honig, B. and Mann, R.S. (2007) Functional specificity of a Hox protein mediated by the recognition of minor groove structure. *Cell*, **131**, 530-543.

17. Blanchet, C., Pasi, M., Zakrzewska, K. and Lavery, R. (2011) CURVES+ web server for analyzing and visualizing the helical, backbone and groove parameters of nucleic acid structures. *Nucleic Acids Res*, **39**, W68-73.

18. Honig, B. and Nicholls, A. (1995) Classical electrostatics in biology and chemistry. *Science*, **268**, 1144-1149.

19. Rocchia, W., Sridharan, S., Nicholls, A., Alexov, E., Chiabrera, A. and Honig, B. (2002) Rapid grid-based construction of the molecular surface and the use of induced surface charge to calculate reaction field energies: applications to the molecular systems and geometric objects. *J Comput Chem*, **23**, 128-137.

20. Kemler, I., Schreiber, E., Muller, M.M., Matthias, P. and Schaffner, W. (1989) Octamer transcription factors bind to two different sequence motifs of the immunoglobulin heavy chain promoter. *EMBO J*, **8**, 2001-2008.

21. Scholer, H.R., Balling, R., Hatzopoulos, A.K., Suzuki, N. and Gruss, P. (1989) Octamer binding proteins confer transcriptional activity in early mouse embryogenesis. *EMBO J*, **8**, 2551-2557.
